# Children’s Picture Interpretation: Appearance or Intention?

**DOI:** 10.1037/a0039571

**Published:** 2015-07-20

**Authors:** Emma Armitage, Melissa L. Allen

**Affiliations:** 1 Centre for Research in Human Development and Learning, Department of Psychology, Lancaster University

**Keywords:** symbolic representation, theory of pictures, artist intention, iconicity

## Abstract

Pictures are defined by their creator’s intentions and resemblance to their real world referents. Here we examine whether young children follow a realist route (e.g., focusing on how closely pictures resemble their referents) or intentional route (e.g., focusing on what a picture is intended to represent by its artist) when identifying a picture’s referent. In 3 experiments, we contrasted an artist’s intention with her picture’s appearance to investigate children’s use of appearance and intentional cues. In Experiment 1, children aged 3–4 and 5–6 years (*N* = 151) were presented with 4 trials of 3-object arrays (e.g., a pink duck, a blue duck, and a teddy). The experimenter photographed or drew 1 of the objects (e.g., blue duck), however, the subsequent picture depicted the referent in grayscale (black and white condition) or the color of its shape-matched object, for example, a pink duck (color change condition). Children were asked 3 questions regarding the identity of the pictures; responses were guided by intentional cues in the black and white condition, but appearance in the color change condition. Experiment 2 confirmed that appearance responses were not due to the artist’s changing knowledge state. Experiment 3 replicated the results of Experiment 1 with adult participants. Together, these studies show that children and adults are neither strictly realist nor intentional route followers. They are realists until resemblance cues fail, at which point they defer to intentional cues.

Visual symbols can be arbitrary, bearing no resemblance to their referent, or iconic, closely resembling what they refer to in the world. Although pictures are often iconic, what a picture looks like is not always sufficient to identify its referent or communicative function (Browne & Woolley, 2001; Myers & Liben, 2012). A long-standing debate in the literature addresses exactly how children develop a theory of pictures and link pictures to real world referents (Callaghan, 2013; Callaghan, Rochat, & Corbit, 2012; DeLoache, 1987, 1995; Freeman, 2000; Gibson, 1954, 1971; Gombrich, 1961; Goodman, 1976; Preissler & Carey, 2004; Wollheim, 1987), as these skills represent significant developmental achievements (DeLoache, 2004). One possibility is that young children are *realists*, deciphering a picture solely in terms of what it looks like. Alternatively, they may rely upon the *intention* of the artist when interpreting the picture–referent relationship. The current set of experiments investigates these two potential picture interpretation strategies in children aged 3–6 and adults.

The realist and intentional strategies can be deduced from Freeman and Sanger’s (1995) intentional net framework, which posits that a theory of pictures is formulated by analyzing relations between four factors: the picture, the artist, the world, and the beholder. The realist strategy privileges the relationship between the picture and the world, while the intentional strategy focuses on the artist-picture relationship. Philosophers adopt divergent positions regarding which of these relationships is more important for picture interpretation (Barthes, 1977; Bazin & Gray, 1960; Dewey, 1958; Ittelson, 1996; Walton, 1984; Wimsatt & Beardsley, 1946). Plato (1987) for instance, advocated the realist perspective, relegating the artist to the role of imitator and denying their ability to communicate anything deeper than that which can be physically seen in their work. Contrastingly, Wollheim (1987) adhered to the intentional picture interpretation strategy. He credits the artist with a pivotal role in the interpretation of his or her work and limits the artwork itself to the role of communicative vehicle, arguing, “if we are interested in . . . paintings, we must start with the artist” (Wollheim, 1987, p. 36). Contemporary developmental studies also reveal conflicting positions regarding this debate.

On one hand, what a picture looks like appears critical to the early understanding of pictures. Highly iconic pictures facilitate generalization of labels from pictures to their real world counterparts (Ganea, Pickard, & DeLoache, 2008; Tare, Chiong, Ganea, & DeLoache, 2010), the imitation of actions seen in a picture book (Simcock & DeLoache, 2006), and the symbolic use of pictures, for instance, using photographs to identify which toys to place in a box (Callaghan, 2000). In some studies, even older children have been shown to consistently focus on a drawing’s appearance despite receiving explicitly contradictory information regarding what it was intended to represent (Browne & Woolley, 2001; Richert & Lillard, 2002). Richert and Lillard introduced 4- to 8-year-old children to Luna, a troll doll who lived in a “land without animals.” After Luna drew something that looked like a fish, children were simply asked if Luna had in fact drawn a fish. It was not until 8 years of age that children considered the mental state of the artist and did not name the pictures based upon their appearance. Of course, children may have misinterpreted the question as asking, “Does Luna’s drawing *look like* a fish?” thus biasing them toward interpreting the picture based upon its appearance. Nevertheless, this finding suggests that children may have difficulty taking intention into account when it conflicts with appearance.

Additional support for the importance of appearance comes from Browne and Woolley (2001, Study 1, Task 1). In their study, a puppet told 4- to 7-year-old children and adults that he was trying to draw a bear, but instead drew something else entirely, such as a rabbit. When asked what the drawing should be named, 84% of 4-year-olds and 94% of 7-year-olds gave appearance-based answers, as did adults, which supports a realist view of picture interpretation.

Other evidence suggests that complete pictorial competence requires a deeper understanding of the complex relationships that “can exist between depiction and reality” (Pierroutsakos & DeLoache, 2003, p. 155). That is, one must consider the artist’s role in shaping a picture’s appearance; an unconscious awareness of which has recently been demonstrated empirically in work with adults (Taylor, Witt, & Grimaldi, 2012). Sensitivity to artist intention has been identified as early as 2.5 years old. Preissler and Bloom (2008) showed that children were able to use the gaze of an experimenter to link a picture to a real world referent, even when the picture could plausibly refer to more than one object. They only did so, however, during an intentional act of drawing, and not when merely associative cues were provided. Furthermore, in a clever study by Bloom and Markson (1998), 3- and 4-year old children were asked to draw pairs of pictures, such as a balloon and a lollipop. Even though these pictures could not be distinguished by shape, children could successfully name them after a brief delay, which suggests that they were using their own intentions to keep track of the identity of their visual depictions. However, while the pictures produced were indistinguishable by shape, they were distinguishable by color. A replication in which color was held constant found that children performed at chance when labeling their drawings (Callaghan & Rochat, 2008), thus suggesting that in the original study children may have relied on color cues rather than their intentions.

Nonetheless, further support for the importance of intentionality for interpreting pictures can be derived from Gelman and Ebeling’s (1998) work. They told 2- to 4-year-old children and adults that a series of pictures had been created intentionally (“John used some paint to make something for his teacher”) or accidentally (“John spilled some paint on the floor”). Children and adults in the intentional condition were significantly more likely to name the drawing according to its shape (i.e., the outline of a man was named “a man”) than participants in the accidental condition, who displayed a trend toward material-based naming (e.g., paint).

The key insight derived from previous literature is that different testing paradigms have given rise to conflicting conclusions about children’s picture interpretation. When pictures are ambiguous children may use the intentional cues provided by the artist-picture relationship to interpret them (Bloom & Markson, 1998; Preissler & Bloom, 2008). However, when a picture’s appearance conflicts with what it was intended to represent (Browne & Woolley, 2001; Richert & Lillard, 2002) it appears that children, and adults, might rely on the picture–world relationship and prioritize appearance cues. Thus, it is critical to combine these distinct methodologies in a single experiment using the same stimuli to determine whether the transparency of the picture–world relationship predicts the use of appearance and intentional cues. This is one aim of our first experiment.

Beyond methodological differences, it is also important to look at the modality of picture production to determine its influence on picture interpretation strategies. Most studies focus upon drawings, the handmade creation of which establishes a clear and salient link between an artist and his or her picture. Photographs provide an interesting comparison, as arguably, the role of the artist is less clear-cut, not least because picture creation is mediated by the mechanics of a camera and printer. In Liben and Szechter (2002) and Szechter and Liben’s (2007) work, 7- to 13-year-old children demonstrated an overwhelming tendency to evaluate, sort, and pair photographs according to their content. These findings fall in line with prior work on drawings, which show that pictures are primarily evaluated on their appearance. However, Liben and Szechter (2002) also found that when critiquing photographs they disliked, around 15% of 7- to 8-year-old children’s comments referred to the photographer’s actions. Although not a significant proportion, this suggests that children have at least some awareness of the importance of the image creator in evaluating photographs. An empirical comparison of photographs and drawings would contribute to a more global understanding of how children develop a theory of pictures.

In Experiment 1, we used condition and modality manipulations to investigate when and how 3- to 6-year-old children use appearance and intentional cues to interpret pictures. In order to explore whether there is a developmental trajectory associated with children’s cue use we used two age groups: 3- and 4-year-old children, who have only recently begun using intentional cues to interpret pictures (Bloom & Markson, 1998; Gelman & Ebeling, 1998; Preissler & Bloom, 2008), and 5- and 6-year-old children, who have a more sophisticated conception of the relationship between artists and their pictures (Callaghan & Rochat, 2003), and a keener understanding of others’ minds (Callaghan et al., 2005; Keysar, Lin & Barr, 2003; Wellman, Cross, & Watson, 2001). For these reasons we anticipated that the older age group would rely more on intentional cues for interpreting pictures than the younger age group. In summary, the current experiment assesses how two distinct age groups of children use intentional cues to interpret pictures, both when intention acts as a solitary cue and when it conflicts with appearance.

Including children of an age where considerable pictorial experience is assumed (5–6 years) also allowed us to investigate whether there were any differences in how the two age groups employ appearance and intentional cues when interpreting photographs compared with drawings. We anticipated that the 3- and 4-year-old children would give fewer intentional responses in the photograph task than the line drawing task because the addition of a camera and printer may make it harder to track how the photographer’s intentions map onto their picture. By contrast, we expected 5- and 6-year-old children to successfully incorporate these mechanical intermediaries into their understanding of the photographer-picture relationship due to additional experience with this modality. Alternatively, given that prior research has indicated that even older children might place more emphasis on appearance than intention when evaluating photographs (Liben & Szechter, 2002; Szechter & Liben, 2007), children in both age groups may give fewer intentional responses in the photograph compared to the line drawing task due to the general perception that photographer’s intentions are of less interpretive value. To explore the effect of modality we used two tasks: a photograph task and a line drawing task, each of which consisted of four trials. In each trial children were introduced to three objects: one target object (e.g., blue duck), a second object varying only in color (e.g., pink duck), and a distractor object (e.g., teddy). In two conditions the appearance of a picture was changed in order to create a conflict between what the picture creator intended to depict and what her final picture resembled. In the color change condition the picture creator intended to depict one object (e.g., a blue duck) yet the final picture clearly resembled a differently colored object (e.g., a pink duck). We predicted that the transparency of the picture-world relationship, combined with children’s early and repeated exposure to the perceptual similarity between pictures and their referents (Ganea, Pickard, & DeLoache, 2008; Simcock & DeLoache, 2008), would facilitate reliance on appearance rather than intentional cues in this condition.

In the black and white condition the picture creator intended to depict a colored object, however, the final picture depicted the object in grayscale. Thus, here the relationship between the picture and the world was much less transparent because the picture could represent *either* of the relevant objects (e.g., a pink or a blue duck). However, the artist’s intention identified one of these objects as the picture’s referent. Here we predicted that children would rely on intentional cues due to their sensitivity to the role of artist intention in picture comprehension (Bloom & Markson, 1998; Gelman & Ebeling, 1998; Preissler & Bloom, 2008) and the lack of clarity offered by the picture’s ambiguous appearance.

Children were asked three test questions. They were asked to name the picture (“what is this a picture of?”), to retrieve the referent object (“can you pass me this”) and to recall what the artist had intended to depict (“what did I mean to take a picture of?”). The first two questions were intended to test the dual representation hypothesis. We expected the first question to focus attention on the picture as an object in and of itself and bias children to answer based upon what the picture looks like, while we anticipated that the second question might highlight the symbolic nature of the picture (see Callaghan, 2000, 2013; Callaghan, Rochat, & Corbit, 2012; DeLoache, 1987, 1991, 2004; DeLoache & Burns, 1994; Dow & Pick, 1992; Jolley, 2008) eliciting an intentional focus from participants. However, it was also noted that if children gave the same answer to both questions, the behavioral question could then serve as a corroborative measure of children’s verbal responding, as it has been suggested that children’s aesthetic understanding can be underestimated due to their inability to verbalize what they know (Bloom, 2004; Jolley, Zhi, & Thomas, 1998). The final question was included as a check that children had not forgotten what the experimenter had told them she intended to draw or photograph.

In Experiment 2, we explored the influence of an additional factor, artist knowledge, on children’s choice of picture interpretation strategy. It was hypothesized that in Experiment 1 the artist’s lack of surprise about her picture’s changing appearance (e.g., a blue duck instead of a pink duck) may have invalidated her earlier intention. Thus, we manipulated the artist’s knowledge about the appearance of her picture and contrasted it with the knowledge of a second experimenter, in order to assess the impact of this variable on children’s use of appearance and intentional cues. Finally, as previous research has found strong similarities in how children and adults interpret pictures (Browne & Woolley, 2001; Gelman & Ebeling, 1998) Experiment 3 assessed whether, in the current paradigm, adults would use appearance and intentional cues in the same way as children.

By manipulating the extent to which pictures resemble their real world referents, and contrasting this with what the picture creator intended to depict, we can identify whether participants prioritize the picture–world or artist–picture relationships when interpreting pictures. Furthermore, we can identify the role that an artist’s knowledge plays in evaluating his or her intention. Together these experiments will contribute a deeper understanding of the order in which children and adults utilize the relationships in Freeman and Sanger’s (1995) intentional net, and the factors that influence their usage.

## Experiment 1

In Experiment 1 we explored whether children think appearance or intention is more important for interpreting pictures. We used a between subjects design; children took part in the photograph *or* the line drawing task and in the color change *or* the black and white condition. In both conditions we changed the appearance of a series of pictures to create conflict between what the picture creator intended to depict and what the picture resembled. In the color change condition the color of the referent was changed (e.g., if the experimenter intended to draw a blue duck, the final picture showed a pink duck) and in the black and white condition the picture appeared in grayscale, rather than color. The modality manipulation also allowed us to examine whether children’s cue use differs for photographs and drawings.

### Method

#### Participants

One-hundred and 51 typically developing children between the ages of 3 and 6 participated in the photograph (*N* = 76) and line drawing tasks (*N* = 75). Children were split into two age groups: 3- and 4-year-olds (*M*_age_ = 46 months; Range = 37–59 months) and 5- and 6-year-olds (*M*_age_ = 71 months; Range = 60–82 months), and two conditions, yielding four experimental conditions (see Table 1). Children were recruited from six primary schools, three nurseries, one holiday play scheme, and the database of the Centre for Research in Human Development and Learning (CRHDL) at Lancaster University. Families were predominantly White and middle class.[Table-anchor tbl1]

#### Apparatus and stimuli

##### Photograph task

**Figure 1 fig1:**
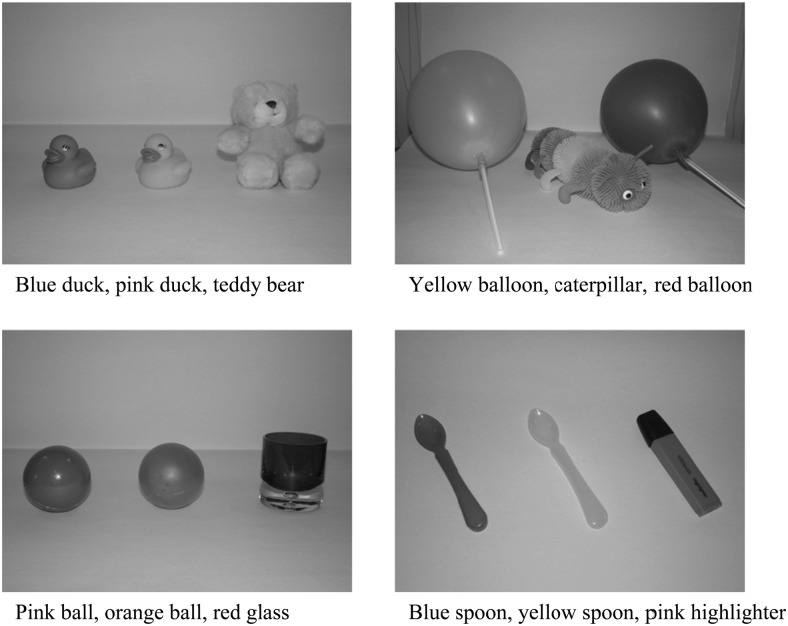
Object arrays.

A 9.1 megapixel Sony digital camera and a HP Photosmart printer were used. Twelve familiar objects arranged into four sets of three object arrays formed the object stimuli. Each array was composed of two test objects and a third distractor object approximately matched in size to the test objects (see Figure 1). Ten color or grayscale photographs (8 in. × 4 in. and presented landscape on A4 photographic paper) of these objects acted as the pictorial stimuli.[Fig-anchor fig1]

##### Line drawing task

Eight coloring crayons and plain A4 paper were used. To permit direct comparisons across tasks the same objects, arranged in the same three-array configurations as in the photograph task were used here. Pictorial stimuli comprised 10 color or grayscale line drawings (8 in. × 4 in. and presented landscape on A4 paper) of these objects.

Design. A 2 × 2 × 2 × 3 mixed design was used. Condition (“color change” and “black and white”), modality (“photograph” and “drawing”), and age group (“3- and 4-year-olds” and “5- and 6-year-olds”) acted as the between-subjects factors. The within-subject factor was question type (“verbal,” “behavioral,” and “memory control”). Intentional responses per question type were summed across trial to form three composite scores (see Coding section).

#### Procedure

##### Photograph task

Children each took part in four trials. In each trial the experimenter set up an array of three familiar objects, drawing attention to each individually (“Oh, look a pink duck, a blue duck, and a teddy bear”). She then photographed one of the objects (“I’m going to take a picture of the blue duck”); the objects were placed sufficiently far apart to ensure it was clear which one was being photographed. The photograph was printed (“Let’s print the picture”), and the children were told, “The printer isn’t working very well today” to provide a plausible reason for the picture printing incorrectly. The printer was set up to simulate printing, but preprinted photographs were loaded into the paper tray ready for the experimenter to retrieve. The participant’s view of the printer was obscured to hide this deception.

In the color change condition, the photograph printed in the color of the shape-matched object from the array. For instance, if the blue duck was photographed, the photograph showed a pink duck. In the black and white condition, the picture printed in grayscale, and thus could plausibly represent both the target and perceptually matched distractor object. Once the picture had “printed,” the experimenter held it up for the child and said, “Oh, look, it printed like this.” To probe for participants’ understanding, we asked three explicit questions about the picture. The verbal question required participants to name the depiction (“What is this a picture of?”), while the behavioral question asked them to provide an overt behavioral response and retrieve the object (“Can you pass me this”). If children responded to the verbal question without using a color term, for example, “duck” the experimenter asked, “which one?” The verbal *and* behavioral questions were included to test the possibility that they tapped different aspects of the dual representation hypothesis (DeLoache, 1987). To ensure this could be adequately explored, question order was counterbalanced to avoid order effects. The memory control question (“What did I mean to take a picture of?”) was included to ensure children correctly understood the artist’s intention and had not forgotten what the experimenter originally took a picture of. This question was always asked last to minimize the risk of biasing the child toward intentional responses to the first two questions.

##### Line drawing task

Children participated in four test trials. The procedure followed that of the photograph task with some minor instructional changes. Children were told the experimenter was going to draw one of the objects (“I am going to draw a picture of the blue duck”). In the color change condition, when choosing a crayon the experimenter looked at the selection (which included both correct and incorrectly colored crayons) and said, “I’m going to use this one” before picking up the “wrong color” crayon. For instance, if the experimenter chose to draw the blue duck, she picked up the pink crayon. In the black and white condition the crayon chosen was always black. Highlighting the creator’s intention to choose a particular marker (in this case, a specific colored crayon) is a method that has been successfully used in previous work to assess children’s understanding of the role intention plays in representation (Myers & Liben, 2008).

#### Coding

In the color change condition, responses were coded as either intentional, appearance, or “other.” Responses were coded as appearance-based if the child’s label for, or physical choice of object, matched the color of the object in the photograph or line drawing. Intentional codes were assigned if the child’s physical object choice or verbal label matched the color of the object initially photographed. The “other” code was reserved for responses that did not conform to either of the above response types, for instance, choosing the distractor object.

In the black and white condition, responses were coded as intentional, nonintentional, or “other.” Intentional codes were assigned if the child’s object choice or label matched the object that was initially photographed. Nonintentional codes were assigned if children’s object choice or label matched the object that was not photographed (e.g., choosing the pink duck when the experimenter had intended to photograph the blue duck). Appearance codes were not utilized here as selecting the nonintended object (e.g., pink duck) did not match the grayscale appearance of the picture. “Other” responses included the distractor object, because this was the only additional response ever provided. The same coding scheme was used for the photograph and line drawing tasks.

### Results and Discussion

To provide an initial view of any patterns in the data, the percentage of question responses falling into each of the three coding categories was calculated. The majority of responses given by participants fell into the “appearance/nonintentional” and “intentional” categories. Children’s responses were coded as “Other,” indicating that they chose the distractor object from the array, on a total of 6.2% of trials. Due to the infrequency of these responses they were removed from subsequent analyses, which focused on comparing appearance/nonintentional and intentional question responses.

Children each had 12 data points, having answered three questions per trial across four trials. Although different stimuli were used on each trial, and trial order was counterbalanced, it was important to check that children’s question responses did not differ as a function of the stimuli used, or the order in which they were presented. McNemar tests were conducted to identify possible stimulus effects, as no such effects were identified the data were collapsed across trials. A repeated measures ANOVA was then used to check for order effects; none were identified. The dichotomous nature of the “appearance/nonintentional” and “intentional” response categories necessitated that only one response type act as the dependent variable. Thus, intentional question responses were chosen and summed across all four trials to provide three composite scores, one per question type: verbal, behavioral, and memory control. The final DV was number of intentional responses out of four trials, thus scores ranged from 0–4. A score of 0 indicated that no intentional responses were given to that question, whereas a score of 4 indicated that intentional responses were given to all questions of that type.

To check whether children had remembered the experimenter’s original intention responses to the memory control question were analyzed first. Those children who responded correctly to the memory control question on three or four trials were placed in the “passed memory control” group, and those who responded correctly on zero, one, or two trials were placed in the “failed memory control” group. A total of 90 children remembered, while 61 forgot (see Table 2 for relevant age and condition groupings). Due to the large number of children who had apparently forgotten the experimenter’s stated intention, responses from children who passed and failed the memory control were analyzed separately using individual 2 (Modality: photograph, line drawing) × 2 (Condition: color change, black and white) × 2 (Age group: 3- and 4-year-olds, 5- and 6-year-olds) × 2 (Question Type: verbal, behavioral) repeated measures ANOVAs.[Table-anchor tbl2]

#### Passed the memory control

**Figure 2 fig2:**
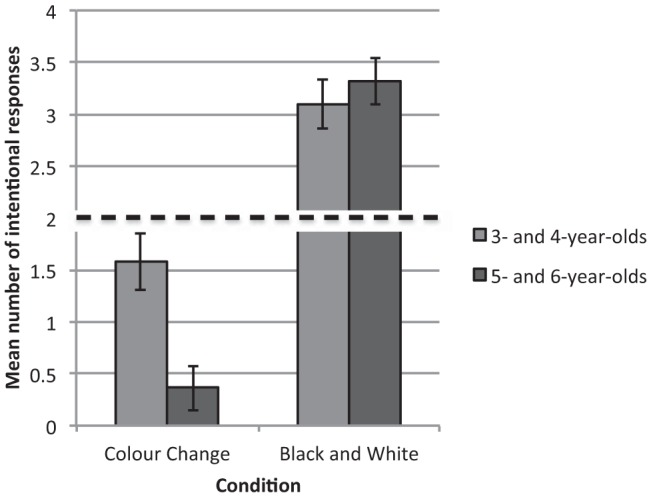
Mean number of Intentional responses given in the color change and black and white conditions by children who passed the memory control.

A significant main effect of question type, *F*(1, 82) = 9.62, *p* = .003, η_p_^2^ = .11, revealed that children gave more intentional responses to the behavioral (*M* = 2.30, *SE* = .13) than the verbal question (*M* = 1.89, *SE* = .14). A significant main effect of modality, *F*(1, 82) = 5.77, *p* = .019, η_p_^2^ = .07, also indicated that children in the line drawing task (*M* = 2.40, *SE* = .16) gave more intentional responses than children in the photograph task (*M* = 1.80, *SE* = .18). Significant main effects of condition, *F*(1, 82) = 87.47, *p* < .001, η_p_^2^ = .52, and age group, *F*(1, 82) = 4.28, *p* = .042, η_p_^2^ = .05, were qualified by a Condition × Age Group interaction, *F*(1, 82) = 9.09, *p* = .003, η_p_^2^ = .10 (see Figure 2). In order to establish the nature of this interaction, additional repeated measures ANOVAs were conducted on data from the two age groups and two conditions separately. These revealed that in the black and white condition, 3- and 4-year-old children (*M* = 3.16, *SE* = .28), *F*(1, 37) = 12.88, *p* = .001, η_p_^2^ = .26, and 5- and 6-year-old children (*M* = 3.31, *SE* = .20), *F*(1, 49) = 114.17, *p* < .001, η_p_^2^ = .26, gave significantly more intentional responses than children in the color change condition (3- and 4-year-olds: *M* = 1.62, *SE* = .32; 5- and 6-year-olds: *M* = .37, *SE* = .19). However, in the color change condition, 3- and 4-year-old children (*M* = 1.58, *SE* = .28) gave significantly more intentional responses than the 5- and 6-year-olds (*M* = .36, *SE* = .22), *F*(1, 47) = 11.79, *p* = .001 η_p_^2^ = .23.[Fig-anchor fig2]

Chance analyses (chance value = 2), conducted using one sample *t* tests, further revealed that in the black and white condition, 3- and 4-year-old children gave significantly more intentional responses to the verbal (*M* = 2.95, *SE* = .33), *t*(21) = 2.87, *p* = .009, and behavioral questions (*M* = 3.36, *SE* = .23), *t*(21) = 5.85, *p* < .001, than would be expected by chance, as did the 5- and 6-year-olds: verbal (*M* = 3.13, *SE* = .32), *t*(23) = 3.51, *p* = .002; behavioral (*M* = 3.50, *SE* = .24), *t*(23) = 6.23, *p* < .001. In the color change condition, 3- and 4-year-old children performed at chance on both the verbal (*M* = 1.35, *SE* = .37), *t*(16) = −1.73, *p* = .10, and behavioral questions (*M* = 1.88, *SE* = .43), *t*(16) = −.28, *p* = .79, while 5- and 6-year-old children gave significantly fewer intentional responses to the verbal (*M* = .22, *SE* = .12), *t*(26) = −14.42, *p* < .001, and behavioral questions (*M* = .25, *SE* = .20), *t*(26) = −7.59, *p* < .001, than would be expected by chance.

#### Failed memory control

A main effect of condition, *F*(1, 53) = 56.14, *p* < .001, η_p_^2^ = .51, revealed that overall children in the black and white condition (*M* = 3.12, *SE* = .16), gave more intentional responses than children in the color change condition (*M* = 1.36, *SE* = .18). One-sample *t* tests confirmed that children in the black and white condition gave more intentional responses than would be expected by chance, verbal: *t*(30) = 4.60, *p* < .001; behavioral: *t*(30) = 6.77, *p* < .001, whereas children in the color change condition gave fewer intentional responses than would be expected by chance, verbal: *t*(29) = −4.57, *p* < .001; behavioral: *t*(29) = −3.80, *p* = .001.

**Figure 3 fig3:**
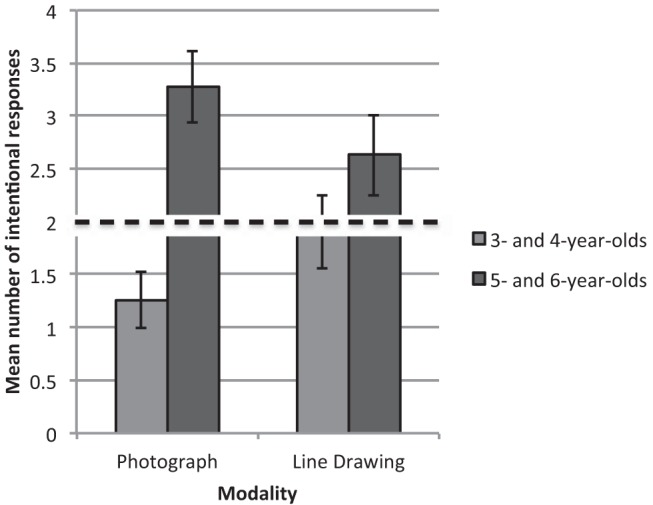
Mean number of Intentional responses given in the photograph and line drawing tasks by children who failed the memory control.

A main effect of age group, *F*(1, 53) = 17.34, *p* < .001, η_p_^2^ = .25, was qualified by a Modality × Age Group interaction, *F*(1, 53) = 6.98, *p* = .011, η_p_^2^ = .12 (see Figure 3). In order to establish the nature of this interaction, additional analyses were conducted on the two tasks and two age groups separately, which revealed that in the photograph task, 5- and 6-year-old children (*M* = 3.27, *SE* = .38) gave significantly more intentional responses than the 3- and 4-year-olds (*M* = 1.26, *SE* = .27), *F*(1, 32) = 21.74, *p* < .001, η_p_^2^ = .41. No such effect was identified in the line drawing task.[Fig-anchor fig3]

In summary, a different pattern of results emerged for children who passed and failed the memory control. Intriguingly, the performance of children in the latter group does not necessarily support the notion that they had forgotten the experimenter’s intention. If these children genuinely did not remember which of the three objects was the intended referent we would have expected them to perform at chance, or to rely consistently on appearance cues when answering the verbal and behavioral questions. Instead, they showed the same bias toward intentional responding in the black and white condition as children who passed the memory control, while the 5- and 6-year-olds in the photograph task also relied on intentional cues to identify the picture’s referent. An alternative explanation is that these children were simply not convinced that the experimenter “meant” to draw or take a picture of the referent she had identified, and thus failed to report her stated intention when asked the memory control question due to its intentional phrasing; “What did the experimenter *mean* to draw a picture of” rather than “What did the experimenter *say* she would draw a picture of?” In other words, while children were unwilling to explicitly endorse the idea that the experimenter had truly intended to draw or photograph one object while producing a picture that contradicted her intention, they were willing to use her statement of intent, in the black and white condition, to identify the picture’s referent.

Returning to those children who did remember the experimenter’s intention, the current findings demonstrate that the referential ambiguity of a picture is of fundamental importance in determining the use of appearance and intentional cues. When an image could represent multiple referents and therefore appearance cues were unavailable, as in the black and white condition, the picture’s identity is ascertained using intentional cues. By contrast, when an image is intended to represent one object but strongly resembles another, as in the color change condition, appearance cues dominate older children’s picture interpretation. Interestingly, 3- and 4-year-old children performed at chance in this condition, indicating that they may be less dependent on the ambiguity of a picture than their older counterparts. As their performance did not exceed chance, intention did not override the picture’s appearance, instead it appears that the younger children were simply less willing to disregard the experimenter’s intention. This may be because they have only recently begun using intentional cues to interpret pictures (Bloom & Markson, 1998; Gelman & Ebeling, 1998; Preissler & Bloom, 2008) and are therefore more attuned to them than older children, who have begun to tailor their cue use to the context in which they encounter pictures. Nonetheless, overall these findings pinpoint picture ambiguity as an important mediating factor in children’s developing picture interpretation.

Beyond the effects of condition and age, children who passed the memory control also gave more intentional responses to the behavioral than the verbal question. This supports the earlier hypothesis that the two questions tap different aspects of dual representation, which refers to the notion that pictures can be thought of as both objects in their own right *and* as representations of other entities. Corroborating our expectations, asking participants to retrieve the picture’s object referent (“Can you pass me this?”) served to remind them of the creator’s intention to depict a specific referent (“I’m going to take/draw a picture of the pink duck”), thereby highlighting the picture’s identity as a symbol that represents something in the real world. By contrast, when asked the verbal question (“What is this a picture of?”), participants could simply look at the picture itself, as an object in its own right, and name it accordingly. Finally, and as predicted, children in the line drawing task gave more intentional responses than children in the photograph task. One explanation for this is that the iconic nature of the photographs focused children’s attention on the picture-world relationship, resulting in an increase in their use of appearance cues and a corresponding decrease in intentional responding. Certainly, prior studies have found that when asked to categorize and evaluate photographs 7- to 13-year-old children largely focus on the visual properties of photographs, mainly content, to the detriment of any in-depth consideration of the photographer’s role in choosing the content or shaping the picture’s appearance (Liben & Szechter, 2002; Szechter & Liben, 2007). In addition, the introduction of a camera and printer may have weakened children’s reliance on intentional cues because the appearance of the final picture is less closely related to the photographer’s intentions, while being clearly linked to the actions of the camera. For instance, technical malfunctions can have unintentional effects on a picture’s appearance, which distort the photographer’s intentions. These two explanations are not mutually exclusive. It is probable that the combination of a stronger picture–world relationship and weaker photographer-picture relationship both contributed to lowering intentional responding. This issue is returned to in the General Discussion.

Having addressed what underlies intentional responding in the black and white condition, it is also important to consider what motivated appearance-based responding in the color change condition. One potentially important extraneous variable in this condition was the artist’s knowledge about the picture. It is possible that when instructing the child that the printer was not working properly (“The printer isn’t working very well today”) in the photograph task, choosing the wrong crayon in the line drawing task, and commenting on the change in the final picture (“It printed like this”) children were misled into thinking that her stated intention was no longer relevant, and consequently that the test questions pertained to the referent depicted in the final picture. This is particularly likely if, as Rosset (2008) states, humans initially interpret all actions as deliberate due to an “intentional bias,” and only develop the ability to override this bias as they gain experience of other explanations for behavior, such as accidental or coincidental events; experience children have relatively little of. Thus, the aim of Experiment 2 was to rule out this explanation as the underlying reason for children’s appearance-based responses in the color change condition of Experiment 1.

## Experiment 2

The aim of Experiment 2 was to clarify the underlying motives for the predominance of appearance responses given to the verbal and behavioral questions by children in the color change conditions of Experiment 1. We hypothesized that these responses may have been prompted by the artist’s failure to comment on the picture’s changing appearance, thereby leading children to believe that her earlier intention was no longer relevant. Previous research has shown that young children are aware that what people see directly affects their knowledge of objects or events (O’Neill, Astington, & Flavell, 1992; Pratt & Bryant, 1990; Pillow, 1989, 1993; Robinson & Whitcombe, 2003; Wimmer, Hogrefre, & Perner, 1988) and can use this information to distinguish between knowledgeable and ignorant observers (Einav & Robinson, 2011; Koenig & Harris, 2007; Robinson, Butterfill, & Nurmsoo, 2011). In order to explore whether the knowledge of the experimenter influenced children’s picture choices in Experiment 1 we added a second experimenter. Experimenter 2 knew what the final picture looked like, but did not know anything about Experimenter 1’s (the artist) intentions. Conversely, while Experimenter 1 knew what she intended the picture to represent she never saw the final image. Half the children were asked the test questions by Experimenter 1, and the other half were asked by Experimenter 2. We expected children to accept that the knowledge of the two experimenters did not overlap, as previous work has shown that much younger children do not expect knowledge acquired by one person to be known by another (Moll, Richter, Carpenter, & Tomasello, 2008; Tomasello & Haberl, 2003). Only the color change condition was used in Experiment 2, as it was in this condition that children were guided by appearance cues.

It was predicted that, if children considered the artist’s knowledge when interpreting the picture, then when Experimenter 1 (the artist) asked the questions, children should give predominantly intentional responses as Experimenter 1 only knew what she intended to depict. When Experimenter 2 asked the questions, children were expected to give appearance responses because Experimenter 2 only knew what the picture looked like. Contrastingly, if the children were staunch realists they were expected to give appearance responses regardless of which experimenter asked the test questions, because the picture’s appearance did not change across conditions. No age group differences were expected.

### Method

#### Participants

Eighty typically developing 3- to 6-year-old children participated. They were split into two age groups: 3- and 4-year-olds (*M*_age_ = 50 months; Range = 40–59 months) and 5- and 6-year-olds (*M*_age_ = 70 months; Range = 60–82 months). See Table 3 for condition and age groupings. Children were recruited from four primary schools in North Yorkshire, and the database of the Centre for Research in Human Development and Learning (CRHDL) at Lancaster University. Families were predominantly White and middle class.[Table-anchor tbl3]

#### Apparatus and stimuli

The materials were identical to the color change condition of Experiment 1; however, due to the nature of the procedure, only two of the original four trials were included. Pilot testing revealed that children would not believe that a second experimenter would interrupt with four “unexpected” phone calls during the short testing session. As no stimulus effects were found in Experiment 1, the duck and spoon trials were randomly chosen.

#### Design

A 2 × 2 × 2 mixed design was used. Condition (“Experimenter 1” and “Experimenter 2”) and age group (“3- and 4-year-olds” and “5- and 6-year-olds”) acted as the between-subjects factors. Question type (verbal and behavioral) was the within-subject factor. For consistency with Experiment 1, intentional responses were summed across trial to form two composite scores, one per question type.

#### Procedure

Children took part in two test trials. As in Experiment 1, on each trial children were introduced to three familiar objects (“Oh, look a pink duck, a blue duck, and a teddy bear”), a photograph was taken of one of the objects (“I’m going to take a picture of the pink duck”) and the photograph was printed. The final pictures always depicted the same object (e.g., duck) but in a different color to that originally photographed (blue duck if the pink duck was photographed) and thus contrasted intention with appearance.

In the Experimenter 1 condition, as the picture printed Experimenter 2 interrupted telling Experimenter 1 there was an urgent phone call for her (“Sorry to interrupt but Melissa is on the phone and she says it is important”). Experimenter 1 left the room to take the phone call, telling the child “I will be back in a minute,” while Experimenter 2 removed the photograph from the printer and showed it to the child (“Wow, this is a nice picture!”). Critically, Experimenter 2 was ignorant of Experimenter 1’s knowledge state and the events that happened until that point, and from the child’s perspective was unaware of which object was actually photographed. Experimenter 1 then ended her phone call, reentered the room and without seeing the final picture, which was held by Experimenter 2, asked the test questions, “What is this a picture of?” (verbal question) and “Can you pass me this?” (behavioral question).

A similar scenario took place in the Experimenter 2 condition, except that after Experimenter 1 reentered the room, Experimenter 2 showed the child the photograph (“Wow, this is a nice picture!”) and asked the test questions, “What is this a picture of?” and “Can you pass me this?” The memory control question, “What did I mean to take a picture of?” was not asked here as it would have been illogical; the first experimenter did not know the picture looked any different to how she intended it to look, and the second experimenter did not know the picture was ever supposed to look any different to how it emerged from the printer. The conflict between these two knowledge states elicited a verbal reaction from several children when the final picture did not resemble the intended object (e.g., informing the second experimenter that, “It was meant to be the pink duck”) and this led to the inclusion of an additional protest measure. Verbal protests have previously been used to assess children’s feelings about social norm violations (Rakoczy, Warneken, & Tomasello, 2008, 2009) and in the current experiment were considered a valuable source of information about how important children considered the artist’s intention. Responses of this type were never given in Experiment 1.

#### Coding

The coding scheme from Experiment 1 was used, with one amendment made. Children’s spontaneous comments regarding the color change in the depicted object were coded as *protests*. For instance, if children said “That’s the wrong color” or “It was supposed to be pink” upon seeing the final picture, this was given a score of 1. Two protest scores were calculated: the number of children who protested was summed, and children were also categorized according to the number of times they protested across the two trials.

### Results and Discussion

Only one question response (out of 320) was coded as “other.” Due to the low frequency usage of this coding category, this response category was removed. As in Experiment 1, intentional responses were used as the dependent variable. McNemar tests revealed no stimulus effects on children’s question responses, thus data were collapsed across trials. Intentional responses were summed to give each child two composite scores, one per question type: verbal and behavioral. Scores ranged from 0–2. A score of 0 indicated that no intentional responses were given to that question type, whereas a score of 2 indicated that intentional responses were given to both questions of that type.

Intentional responses were analyzed using a 2 (Condition: Experimenter 1, Experimenter 2) × 2 (Age Group: 3- and 4-year-olds, 5- and 6-year-olds) × 2 (Question Type: verbal, behavioral) repeated measures ANOVA. No significant main effects or interactions were identified. However, children gave significantly fewer intentional responses than would be expected by chance (chance value = 1) to both the verbal (*M* = .14, *SE* = .05), *t*(79) = −17.42, *p* < .001, and behavioral questions (*M* = .20 *SE* = .06), *t*(79) = −13.95, *p* < .001, indicating a strong reliance on the realist picture interpretation strategy. This replicates the findings of the color change condition in Experiment 1; in the color change condition, when pictures unambiguously resemble a single referent, children rely on appearance cues.

Finally, analysis of the “protest” data revealed that 31/64 (48%) of children protested, and 19/64 (29%) children protested multiple times, demonstrating that they had noticed that the printed picture did not resemble the intended referent. Protests were made equally across the two conditions. Children were split into protesters and nonprotesters and a one-way ANOVA revealed no significant difference in the number of intentional responses given by the two groups; those who protested did not give more intentional responses than those who did not protest. Thus, while the children who protested considered intentional information relevant enough to be noted, they did not consider it to be the “correct” response to the test questions.

Experiment 2 confirmed that realist responding in the color change condition of Experiment 1 was underpinned by a genuine preference for interpreting pictures according to their appearance, and was not influenced by the experimenter’s verbal statements, actions, or knowledge about picture production or the final image. The protests made by children demonstrate an awareness of the conflict between intention and appearance, and in the context of Freeman and Sanger’s (1995) framework indicate that children are spontaneously trying to incorporate multiple pictorial cues into their picture interpretation. By giving appearance-based responses while noting the relevance of intentional cues via verbal protests, children are beginning to demonstrate a sophisticated understanding of the multifaceted nature of picture interpretation. However, given the overall lack of age effects found in Experiments 1 and 2 this raises the question of whether adults use appearance and intentional cues in the same way as children. Experiment 3 addresses this issue by replicating Experiment 1 with adult participants.

## Experiment 3

Previous research has documented that adults and children respond similarly when asked to name ambiguous line drawings (Browne & Woolley, 2001) or pictures produced intentionally versus accidentally (Gelman & Ebeling, 1998). Experiment 3 investigated whether, using the current paradigm, adults would replicate children’s appearance and intentional responding.

### Method

#### Participants

Sixty-four adults (range: 18–52, *M*_*age*_ = 20 years) participated in a replication of Experiment 1 (color change condition: *N* = 32; black and white condition: *N* = 32). They were recruited using opportunity sampling in the North Yorkshire area and via the SONA research participation system at Lancaster University.

#### Apparatus and stimuli

The stimuli from Experiment 1 were used.

#### Design

A 2 × 2 × 3 mixed design was used. Condition (“color change” and “black and white”) and modality (“photograph” and “drawing”) acted as the between-subjects factors and question type (“verbal,” “behavioral,” and “memory control”) as the within-subject factor. Intentional responses per question type were summed across trial to form three composite scores (see Coding section of Experiment 1).

#### Procedure

Prior to the commencement of the experiment, adults were informed that the task had been designed for children and, as such, they should answer based on their intuitions. This was necessary as pilot testing indicated that adults often questioned the nature of the procedure; it is unusual to have someone tell you they intend to draw a particular picture, and then to immediately draw a different one. All other aspects of the procedure followed that of Experiment 1.

#### Coding

The coding scheme from Experiment 1 was used.

### Results and Discussion

An initial exploration of the data revealed that although none of the adults ever chose the distractor object from the array, overall, 7% of their responses were coded as “other.” These responses were largely confined to the black and white condition of the line drawing task and can be split into two categories. Twenty percent (38/192) of these responses involved adults naming the grayscale images according to their final appearance (e.g., gray duck) and thus refusing to choose a target object from the array due to the absence of a gray referent. Here adults were focusing on the picture alone and ignoring the picture-referent relationship; as there was no gray duck in the array, the picture could not represent a gray duck if one was using this relationship. The most likely explanation for such responding is that adults did not believe the experimenter could draw a gray duck when she intended to draw a pink duck, and thus inferred that she must have intended to draw a gray duck. Browne and Woolley (2001) reported a similar finding; 75% of their adult participants attempted to reconcile conflicting appearance and intention cues by stating that ambiguous pictures (e.g., rabbit-bear) looked like their intended referents (e.g., rabbit) rather than the nonintended referents. Together, these findings support Bloom’s (1996) intentional-historical account of artifact concepts, which argues that appearance can be used to infer a picture creator’s intention.

The remaining “other” responses, which accounted for 10% (19/192) of the black and white condition responses, involved adults claiming that the black and white drawings represented both objects (e.g., the pink duck and the blue duck). This gives rise to two potential explanations. First, adults may have been using appearance and intentional cues as equally viable indicators of what the pictures represented, for instance, “it was intended to represent the pink duck, but looks equally like the blue duck, therefore it is a representation of both the pink and blue ducks.” A compatible explanation is that adults’ knowledge of pictorial conventions, specifically that grayscale images are more abstract or generic representations than color pictures (Gelman, Chesnick & Waxman, 2005), allowed them to treat the grayscale pictures in the current study as representations of categories and not specific referents. For instance, a black stick figure represents the category of “men,” not a specific man. In the current experiment, given the absence of a gray duck in the object array adults might have assumed that a black and white picture of a duck represented the two duck shaped objects that were present in the array.

For consistency with Experiment 1 the dependent variable was the number of intentional responses. McNemar tests revealed no stimulus effects on participants’ responses, thus data was collapsed across trials to provide three composite scores, one per question type. Scores ranged from 0–4. A score of 0 indicated that no intentional responses were given to that question, whereas a score of 4 indicated that intentional responses were given to all questions of that type. Unlike the children in Experiment 1, all but two adults correctly recalled the experimenter’s intention on all four trials (the remaining two adults did so on three fourths of the trials). As such, and for consistency with Experiment 1, the memory control question was not included in further analyses.

Intentional responses were analyzed using a 2 (Modality: photograph, drawing) × 2 (Condition: color change, black and white) × 2 (Question Type: verbal, behavioral) repeated measures ANOVA. There was no significant effect of modality. A significant main effect of condition, *F*(1, 60) = 173.44, *p* < .001, η_p_^2^ = .74, revealed that adults in the black and white condition (*M* = 3.84, *SE* = .18) gave significantly more intentional responses than adults in the color change condition (*M* = 0.56, *SE* = .18), indicating that picture ambiguity facilitated adults’ as well as children’s intentional responding. Chance analyses (chance value = 2) further revealed that adults in the black and white condition gave significantly more intentional responses to the verbal (*M* = 3.81, *SE* = .09, *t*(31) = 19.16, *p* < .001, and behavioral questions (*M* = 3.88, *SE* = .06), *t*(31) = 31.57, *p* < .001, than would be expected by chance. By contrast, adults in the color change condition gave significantly fewer intentional responses to the verbal (*M* = 0.56, *SE* = .24), *t*(31) = −6.06, *p* < .001, and behavioral questions (*M* = 0.56, *SE* = .24), *t*(31) = −6.06, *p* < .001, than would be expected by chance.

The findings of Experiment 3 largely replicate those of Experiment 1. Adults follow an intentional strategy to picture interpretation when they are presented with ambiguous pictures, which could represent multiple referents, but switch to realist responding when shown pictures that unambiguously represent a single real world referent. Experiment 3 also extends the findings of Experiment 1 to reveal that adults, unlike children, are aware that when a picture is ambiguous both appearance and intentional cues can identify what it represents; a grayscale image can represent its intended referent (e.g., pink duck) but also any object similar in appearance (e.g., blue duck). Overall, children and adults use appearance and intentional cues similarly when interpreting pictures. However, adults demonstrated a more sophisticated notion of how the two cues interact when pictures are ambiguous, which is likely the result of additional experience with pictures.

## General Discussion

Pictures share both a resemblance-based link to their real world referents, and an intentional link to their creator. The current experiments examined under what conditions children and adults use these cues to interpret pictures. We expected that when the picture–world relationship was transparent, participants would rely on resemblance cues to interpret them, whereas when the picture-world relationship was unclear, they would turn to the artist–picture relationship for intentional cues. In accordance with these hypotheses, we found that children and adults gave predominantly appearance-based responses when asked to name or retrieve the referents of nonambiguous pictures, while relying on intentional cues when interpreting ambiguous pictures. In addition, picture modality and question type exerted an important influence on children’s use of intentional cues. In theoretical terms, our findings suggest that picture–world relations are prioritized over artist–picture relations (Freeman & Sanger, 1995).

Experiments 1 and 3 revealed that overall children and adults identified unambiguous pictures, which resembled only one object referent (color change condition), as representing the referent they resembled and not the intended referent. In line with our hypotheses, this indicates that when the picture–world relationship is transparent, resemblance cues are prioritized over intentional cues, which suggests that both children and adults have a predisposition to judge pictures based on the extent to which they resemble their real world referents (Browne & Woolley, 2001; Richert & Lillard, 2002). The realist responding of adults, and children who passed the memory control, cannot be attributed to forgetting the artist’s stated intention, because they exhibited a high level of intentional responding to the memory control question.

It is important to note that children who failed the memory control also employed appearance and intentional cues differently across the two conditions, only relying on appearance cues in the color change condition. This provides strong evidence that these children did remember the experimenter’s intention, since they too used picture ambiguity to inform their cue use, and suggests that the reason they did not pass the memory control was because they did not think the experimenter “meant” to draw or photograph the referent she had identified as the target. The intentional phrasing of the memory control question, “What did I *mean* to draw a picture of?” combined with children’s skepticism regarding the veracity of the experimenter’s intention, may have made them less likely to give intentional responses to this question. By contrast, when asked the verbal (“What is this a picture of?”) and behavioral (“Can you pass me this?”) questions, which did not require children to believe the experimenter’s intention, those who failed the memory control did rely on her statement of intent to identify the picture’s referent, in the black and white condition. Consequently, it would be interesting for future research to compare responses to two different versions of the memory control question: “What did I *mean* to draw a picture of?” and “What did I *say* I would draw a picture of?” If children disregard intention only when they are asked to agree that the experimenter *meant* to draw, for instance, a pink duck, but not when they are asked to confirm that she *said* she would draw a pink duck, this would confirm that the intentional phrasing of the question used in the current experiments, rather than genuine forgetting, was responsible for the high number of those who failed the memory control.

Experiment 2 also ruled out the possibility that children’s realist responses in the color change condition were a reaction to the artist ignoring that the picture’s appearance had changed. They gave realist responses regardless of whether the experimenter knew what the picture was intended to represent or not, apparently confirming that they were genuinely focusing on the picture’s appearance. However, despite giving overwhelmingly realist responses to the test questions, 48% of children spontaneously acknowledged the importance of the artist’s intention by making one or more attempts to inform the experimenter that the picture’s appearance was “wrong” or unexpected in relation to the original intention (e.g., “She took a picture of the blue one, and now it is pink”). This suggests that children were processing appearance and intention cues in parallel. While appearance responses were dominant, children were aware of intentional information and made a conscious effort to ensure it was not ignored. There is another potential explanation for children’s protests, which is that they simply did not like the discordant knowledge of the two experimenters and were attempting to resolve the conflict. While this is plausible, the work of Rakoczy and colleagues supports our initial claim; they report that children protest when a new player violates the rules of a game, precisely because they know that the rules are important (Rakoczy, Warneken, & Tomasello, 2008, 2009). Previous research has shown that children are aware that an artist’s intention is important when interpreting pictures (Gelman & Ebeling, 1998; Preissler & Bloom, 2008; Richert & Lillard, 2002), and thus in the current experiment, when children realized that the experimenter’s intention had been violated without her knowledge they protested to ensure that, at the very least, the experimenter knew that he or she understood the relevance of the intentional information for interpreting the picture.

The realist bias found in the present experiments can be explained with reference to the pictorial experience of children and adults. Children’s picture books are typically made up of pictures that clearly resemble their real world referents, and adults talk to children about the link between these pictures and the world by labeling them (Fletcher & Reese, 2005), and pointing out their relevance to the child’s own world using statements such as, “Jelly, you had jelly on your toast this morning” (DeLoache & DeMendoza, 1987, p. 114). Thus, children learn that pictures represent the world by virtue of resemblance, and from the age of 15 months iconicity facilitates their ability to map information from pictures to their real world referents (Callaghan, 2000; Chiong & DeLoache, 2013; Ganea, Pickard, & DeLoache, 2008; Tare, Chiong, Ganea, & DeLoache, 2010). Perceptual similarity enhances the transparency of the picture–referent relationship and therefore makes it easier for children to understand that one refers to the other.

Children are also encouraged to make their own pictures recognizable. Adults often ask young children to name their scribbles and these label requests reinforce resemblance as a defining characteristic of pictures. Furthermore, Callaghan (1999) found that 3- and 4-year-old children make their drawings more recognizable after they are told that an adult cannot match their picture to its referent. Intense focus on what a picture looks like persists until the age of around 11 or 12, with children typically referring to the appearance or content of a picture when asked their opinion (Parsons, 1987), and using subject matter to evaluate aesthetic beauty (Freeman & Sanger, 1995). Thus, the majority of children’s early pictorial experiences revolve around transparent picture–world relationships that can be understood via resemblance. Consequently, when faced with unambiguous pictures in the color change condition of the present experiments, children relied on resemblance cues because that is how they are familiar with interpreting pictures.

Experience may also be implicated in adults’ realist responding, however, there is another explanation that might account more adequately for their performance. It is possible that adults might not have believed that the experimenter could reasonably intend to draw or photograph one object and instead produce a picture of a different object. Previously it has been found that adults have strong expectations about the correspondence between pictures and their referents, namely that they should look like one another (Browne & Woolley, 2001). This expectation may have encouraged adults to try and resolve the cue conflict by inferring intention from appearance (Bloom, 1996; Bloom & Markson, 1998), for instance, “She must have intended to draw the blue duck because that is what her picture looks like.” Alternatively, they may have tried to decipher the pragmatics of the situation and ultimately, decided that appearance was a more stable cue given the inconsistent nature of the intentional cues.

Despite the staunch realism found in the color change condition of the current experiments, we also found that children and adults appreciate that what an artist intends to depict is an important determinant of what a picture represents (Wollheim, 1987). In line with our hypotheses, visually ambiguous pictures, those that equally resembled two object referents (black and white condition), were identified as representing their intended referent. This finding supports the claim that children are sensitive to the intentional cues provided by a picture creator from an early age (Gelman & Ebeling, 1998), but also confirms that intention is only prioritized when the picture’s appearance is insufficient to determine its referent (Bloom & Markson, 1998; Browne & Woolley, 2001; Preissler & Bloom, 2008).

Children and adults’ ability to disambiguate pictures using an artist’s intention fits into a wider body of literature concerning how attuned humans are to intentionality. Between 14- and 18-months-old, children begin to infer intentionality from failed actions (Meltzoff, 1995), eye gaze and pointing (Behne, Carpenter, & Tomasello, 2005; Liebal, Behne, Carpenter, & Tomasello, 2009), and by 2.5 years old, children can infer an artist’s intention from his or her eye gaze (Preissler & Bloom, 2008). Together, this suggests a natural proclivity for intentional information, which is further supported by studies showing that adults are unconsciously biased toward intentional explanations for behavior (Rosset, 2008). This raises the question of why artist intention seems to function as a secondary cue to picture interpretation, when philosophers argue that it is a defining feature of what a picture represents (Barthes, 1977; Gombrich, 1972; Goodman, 1976; Scruton, 1981; Wollheim, 1987) and psychologists consider it to play a crucial role in the communicative efficacy of pictures (DeLoache, 2004).

One of the reasons children may not immediately use intentional cues to interpret pictures, is that they lack experience of doing so. It is uncommon for children to receive explicit instruction regarding how artists relate to their pictures. Picture book interactions typically consist of adults asking children to identify pictures (e.g., “What is it?”) or report something about the depicted content, such as the sound a snake makes, (DeLoache & DeMendoza, 1987; Gelman, Chesnick, & Waxman, 2005) rather than, “Who do you think made this picture?” or “What do you think the person was trying to draw?” This lack of experience coincides with the fact that in everyday life people are not required to use intentional information to interpret pictures because they typically resemble what their artists intend them to (Bloom, 1996), meaning appearance-based responses are often sufficient. Although children are reluctant to spontaneously refer to an artist’s role in picture production (Gardner, Winner, & Kircher, 1975), when it is explicitly demonstrated or intentions are stated, as in most research paradigms (Browne & Woolley, 2001; Callaghan & Rochat, 2003; Preissler & Bloom, 2008; Richert & Lillard, 2002), children can and do utilize intentional cues as they did in the black and white condition of Experiment 1.

Overall, there was no difference in the performance patterns of children and adults; however, adults’ responses to the black and white condition do provide room for conjecture regarding how their approach to the task may have differed from that of children. Adults in the line drawing task displayed a tendency to name the grayscale picture according to its final appearance (e.g., gray duck) or to state that the picture represented both target objects (e.g., pink and blue duck). The latter response type suggests that adults were either combining appearance and intentional cues (e.g., it was meant to be a pink duck but looks equally like both ducks) or assuming that the grayscale image served as a representation of a category, “ducks,” rather than of a specific exemplar. Together, these responses suggest that the considerable experience adults have of using pictures as symbols allowed them to approach the current task with greater representational flexibility than children. This experience also imbues them with the knowledge that picture interpretation is one domain in which beholders have the power to construct their own subjective interpretations without being “wrong” (Freeman & Sanger, 1995; Gombrich, 1961; James, 1890/1950; Wollheim, 1987). This manifested itself in adults combining cues that were presented individually, as well as going beyond the provided cues to apply their broader knowledge of how pictorial conventions function in the real world, for instance, approaching black and white pictures as generic representations.

The knowledge that we can interpret the world in multiple ways, and that people can perceive the same picture differently (Lagattuta, Sayfan, & Blattman, 2010) is referred to as an interpretive theory of mind (iToM). The onset of iToM is around the age of 7 (Carpendale & Chandler, 1996; Chandler & Helm, 1984; Taylor, 1988), as children in the present study were aged between 3 and 6 this explains why in the present experiment adults felt confident in manipulating the responses explicitly provided by the task, while children never did this. Future research could explore whether older children who have acquired an iToM might manipulate their responses as adults did, thereby demonstrating a developing insight into the subjective nature of pictorial representations.

We also explored the dual representation hypothesis by asking two different questions to tap into children’s perception of pictures as symbols or as concrete objects. As was hypothesized, asking children to name the picture focused attention on the picture as an object in its own right (DeLoache, 1991), whereas, asking them to retrieve the picture’s object referent highlighted the picture-as-symbol aspect of dual representation. At the heart of symbolization is the intention to communicate, and thus children were reminded of the artist’s intention to depict a specific referent (“I want to take/draw a picture of the pink duck”), thereby increasing intentional responding. This extends previous research by showing that children’s picture interpretation is influenced by how they are asked to think about the picture—in isolation, or as a symbol that represents a specific real-world referent—as well as the cues provided by the picture itself. Those studies that have reported strong realist responding have typically only asked participants to name pictures or to report what they look like (Browne & Woolley, 2001), which may have biased them toward focusing solely on the picture’s appearance at the expense of a broader approach taking into account the artist’s input.

Modality was the final manipulation used in Experiments 1 and 3. It was anticipated that children would rely less on intentional cues in the photograph task compared with the line drawing task, as prior work has shown that children place little emphasis on the intentions of photographers, preferring to focus on referential content when asked to discuss specific photographs, or the process of photography (Kose, 1985; Liben & Szechter, 2002; Szechter & Liben, 2007), yet they use artist’s intentions to name drawings across a variety of paradigms (Browne & Woolley, 2001; Gelman & Ebeling, 1998; Preissler & Bloom, 2008). In support of these hypotheses, children in the photograph task who passed the memory control gave fewer intentional responses, across both conditions, than children in the line drawing task. Nonetheless, intentional responding was not at floor level, suggesting that children did not entirely disregard the photographer’s intention, which is in line with Liben and Szechter’s (2002) finding that 7- to 8-year-old children make at least some references to the photographer’s actions when they are asked to critique photographs they dislike. If children are not entirely unaware of the relevance of the photographer’s intention, the more important question is why photographers’ intentions are devalued relative to those of artists?

Children begin to use verbal and nonverbal cues, such as eye gaze, to infer an artist’s intentions and decode drawings between the ages of 2.5 and 3 years (Bloom & Markson, 1998; Gelman & Ebeling, 1998; Preissler & Bloom, 2008). Associative cues are not sufficient for this mapping to occur (Preissler & Bloom, 2008), thus indicating that young children are knowledgeable about the criteria for using intentional cues to interpret drawings. The link between intention and photography is less visible; although the photographer also gazes at a referent or scene, the presence of the camera and reliance on a printer (in our experiment) in order to produce the final output could disrupt children’s developing ability to map a photographer’s intentions directly onto their pictures, while simultaneously reinforcing the idea that photographs depend on cameras and their real world referents.

Scant research on children’s understanding of the causal-mechanical nature of photography exists to confirm this claim, however, Wellman and Hickling (1994) reported that when they asked 6-, 8- and 10-year-old children to explain how instant photographs are made, approximately 70% of 6-year-olds gave answers such as “the camera gets the idea of it and draws the picture” (p. 1572), indicating that they attribute all decision-making to the camera, rather than the photographer. It is an open question whether the recent increase in young children’s use of camera-enabled devices and photograph-related apps (Rideout, Saphir, Pai, Rudd, & Pritchett, 2013) will rectify or reinforce this understanding of photography, but at the very least, the introduction of a mechanical intermediary weakens the assumption that the creator’s intention determines what a picture represents, and at worst, facilitates the perception of photography as a causal process in which the creator’s intention exerts minimal influence (Bazin & Gray, 1960; Black, 1979; Bloom, 1996; Browne & Woolley, 2001; Costello & Phillips, 2009; Schier, 1986). Work by Browne and Woolley (2001, Study 1, Task 3) provides support for this argument. When they introduced children to a puppet named George who wanted to draw a picture of a cow, yet “accidentally” traced a picture of a horse, without seeing the final picture, 7-year-olds and adults named the drawing a horse, the referent it was causally linked to, ignoring George’s intention to draw a cow. These findings indicate that for older children causality usurps intention, and the current results suggest that younger children may take a similar view.

Importantly, this latter explanation would predict that adults too should have given fewer intentional responses in the photograph than the line drawing task, which they did not. This might be due to the simplicity of the experimental paradigm. In everyday life photographers’ intentions are typically thwarted by a referent moving or a camera malfunction; because neither of these things occurred in the current study, and because the change in the picture’s appearance could be attributed to a printer error, our adult participants may not have perceived the photographer’s intention as being any less valid than the artist’s intention in the line drawing task. Clearly, additional research is needed to identify why intention may be a less valued cue in the medium of photography than drawing, and for adults this may need to consist of a paradigm in which the photographer’s intentions are more realistically disrupted. Nonetheless, our study is the first to directly compare drawing and photography and show that intentional cues are utilized less in the latter.

One final finding from the modality manipulation was that the 5- and 6-year-old children who failed the memory control gave more intentional responses in the photograph task than the 3- and 4-year-olds. In the photograph task it was possible for children to reconcile the conflict between appearance and intention by attributing the change in the picture’s appearance to a printer error, thereby validating the experimenter’s intention. Thus, it may be that even though these children were generally skeptical of the experimenter’s intention they found it to be most plausible in the photograph task, leading to a greater number of intentional responses. The younger children did not make this same judgment. This may be because they had genuinely forgotten the experimenter’s intention, although this is unlikely, as they too relied on intentional cues in the black and white condition. It is more plausible that the younger children simply did not attribute the picture’s appearance to the printer error, meaning the conflict between appearance and intention was not lowered.

The present studies focused on color iconicity; however, color is only one of the ways in which symbols resemble their referents. Future work should therefore investigate the importance of appearance and intentional cues for other types of iconicity, such as shape, form, and size (see Sloutsky, 2003 for a discussion of the relative importance of different similarity relations). For instance, if the printer had produced a crocodile instead of a duck we anticipate that appearance-based responding would have increased, because shape is a powerful cue in defining an object’s identity (Bloom & Markson, 1998; Gelman & Ebeling, 1998; Landau, Smith, & Jones, 1988; Landau, Smith, & Jones, 1998), and thus may override the importance of a creator’s intention to a greater extent than was seen in the current experiments. Certainly, when Browne and Woolley (2001) presented their participants with a drawing that was intended to represent a bear, but looked like a rabbit, both children and adults were swayed by its appearance and named it “a rabbit.” By contrast, size might have had a weaker impact on intentional responding. A picture of a pink duck, which depicts the duck as larger or smaller than it is in reality does not contradict the artist’s intention (to depict a pink duck) as strongly as a differing shape might; it remains a representation of a pink duck. As the present results are specific to color iconicity it is important for future research to build upon our findings by addressing how powerful other resemblance-relations are in terms of overriding intentional cues when interpreting pictures.

The current experiments provide a unique contribution to the pictorial development literature by combining distinct methodologies from extant studies to reveal that the transparency of the picture–world relationship can predict the use of appearance and intentional cues, with the latter dominating only when pictures are ambiguous. In addition, by incorporating age, modality, and question type manipulations, differences were identified in the extent to which intentional cues are used to interpret drawings compared to photographs. Ultimately, this yielded several important insights into the relative importance that is assigned to the various factors that impact picture interpretation.

Taken together, our findings have theoretical implications for Freeman and Sanger’s (1995) intentional net. The relationships within the net appear to be processed hierarchically. The picture–world relationship is attended to first, and if it is insufficient to provide a clear picture interpretation participants utilize the artist–picture relationship as an additional source of information. Furthermore, modality and questioning inform children’s use of appearance and intentional cues. This extends previous work (Bloom & Markson, 1998; Browne & Woolley, 2001; Freeman & Sanger, 1995; Gelman & Ebeling, 1998; Preissler & Bloom, 2008; Richert & Lillard, 2002) by showing that children and adults are not realists *or* intentional picture interpreters; rather they adapt their cue use to fit the specific picture they are viewing, and the context in which they are asked to think about it.

## Figures and Tables

**Table 1 tbl1:** Number of Children in Each Age Group per Task and Condition

	Photograph		Line drawing	
	Color change	Black and white	Color change	Black and white
3- and 4-year-olds	20	18	18	19
5- and 6-year-olds	17	21	19	19

**Table 2 tbl2:** Number of Children Who Passed and Failed the Memory Control According to Age Group and Condition

	3- and 4-year-olds		5- and 6-year-olds	
	Color change	Black and white	Color change	Black and white
Passed	17	22	27	24
Fail	21	15	9	16

**Table 3 tbl3:** Number of Children in Each Age Group per Condition

	Experimenter 1	Experimenter 2
3- and 4-year-olds	18	15
5- and 6-year-olds	25	22
